# Dialogue between mitochondria and endoplasmic reticulum-potential therapeutic targets for age-related cardiovascular diseases

**DOI:** 10.3389/fphar.2024.1389202

**Published:** 2024-06-13

**Authors:** Chen Chen, Xueyan Dong, Wang Zhang, Xing Chang, Wulin Gao

**Affiliations:** ^1^ First Clinical Medical College, Shandong University of Traditional Chinese Medicine, Jinan, China; ^2^ Department of Hematology, Affiliated Hospital of Shandong University of Traditional Chinese Medicine, Jinan, China; ^3^ Shandong Provincial Mental Health Center, Jinan, China; ^4^ Guang’anmen Hospital, China Academy of Chinese Medical Sciences, Beijing, China; ^5^ Department of Geriatric Medicine, Affiliated Hospital of Shandong University of Traditional Chinese Medicine, Jinan, China

**Keywords:** mitochondria-associated endoplasmic reticulum membranes, aging, cardiovascular diseases, mitochondrial bioenergetics, calcium homeostasis

## Abstract

Mitochondria-associated endoplasmic reticulum membranes (MAMs) act as physical membrane contact sites facilitating material exchange and signal transmission between mitochondria and endoplasmic reticulum (ER), thereby regulating processes such as Ca^2+/^lipid transport, mitochondrial dynamics, autophagy, ER stress, inflammation, and apoptosis, among other pathological mechanisms. Emerging evidence underscores the pivotal role of MAMs in cardiovascular diseases (CVDs), particularly in aging-related pathologies. Aging significantly influences the structure and function of the heart and the arterial system, possibly due to the accumulation of reactive oxygen species (ROS) resulting from reduced antioxidant capacity and the age-related decline in organelle function, including mitochondria. Therefore, this paper begins by describing the composition, structure, and function of MAMs, followed by an exploration of the degenerative changes in MAMs and the cardiovascular system during aging. Subsequently, it discusses the regulatory pathways and approaches targeting MAMs in aging-related CVDs, to provide novel treatment strategies for managing CVDs in aging populations.

## 1 Introduction

According to the 2019 Global Health Estimates by WHO, cardiovascular disease has remained the leading cause of death worldwide over the past 20 years. Being strongly correlated with age, the morbidity and mortality rates of CVDs continue to increase as the global population ages. In 2019 alone, there were an estimated 523.2 million new cases and 18.6 million deaths attributed to CVD worldwide (Jenkins et al., 2021). During the aging process, the structure and function of the heart undergo age-related degenerative changes, while various cells may experience pathological changes such as mitochondrial dysfunction, endoplasmic reticulum stress, and oxidative stress due to external adverse factors. These changes significantly increase the risk of CVDs, such as hypertension, atherosclerosis, myocardial infarction, and heart failure (Lakatta and Levy, 2003).

Normal mitochondrial function is essential for energy-demanding systems, such as the cardiovascular system, serving not only as energy providers, but also heavily involved in environmental stress adaptation and tissue function regulation, including metabolism, inflammation, calcium regulation, and cell death (Lopez-Crisosto et al., 2017). Consequently, mitochondria, being highly dynamic organelle networks, are strictly controlled by mitochondrial quality control mechanisms (MQC), with damaged or dysfunctional mitochondria being eliminated through mitochondrial autophagy degradation. When the mitochondrial autophagy mechanism malfunctions, damaged mitochondria accumulate in myocardial cells, vascular smooth muscle cells (VSMCs), and endothelial cells (ECs), triggering inflammatory reactions, and potentially leading to myocardial cell death and tissue damage (Amgalan et al., 2017; Gisterå and Hansson, 2017). Consequently, MQC disorder can promote the pathogenesis of CVDs, such as ischemia/reperfusion, hypertension, atherosclerosis, heart failure, etc. The aging process of the heart is accompanied by a general decline in mitochondrial function, the release of MQC, and the loss of mitochondrial ER interaction, which are closely associated with age-related CVDs (Li et al., 2019).

The endoplasmic reticulum (ER), as the largest multifunctional organelle, hosts various subcellular compartments crucial for numerous cellular physiological processes. These include protein secretion, folding, and transport; intracellular Ca^2+^ uptake, storage, and signaling, among others (Namba, 2015; Schwarz and Blower, 2016). Dysfunction of normal ER function can result in the accumulation of unfolded and misfolded proteins, leading to ER stress and potentially cell apoptosis. This process is associated with pathological conditions such as ischemia, hypoxia, oxidative stress, and depletion of Ca^2+^ stored in the ER (Ma and Hendershot, 2004). Relevant studies have found that ER stress is an important regulator of CVDs and is closely related to ischemic heart disease, hypertension, and heart failure (Zhou et al., 2021). Due to the gradual decline in the buffering capacity of the proteostasis network with aging, there is an accumulation of intracellular aggregates comprising misfolded proteins, leading to alterations in the ER environment in diseases such as cardiovascular disorders, thereby disrupting protein homeostasis (Arrieta et al., 2020). Loss of protein homeostasis and subsequent accumulation of unfolded and misfolded proteins are central molecular hallmarks of aging and many degenerative diseases.

Recent studies have revealed not only functional interaction but also physical connection between mitochondria and the ER. The connection site is known as mitochondria-associated endothelial reticulum membranes (MAMs) or mitochondria ER contact sites (MERCs). Through functional and physical interactions, MAMs jointly regulate cell mitochondrial homeostasis, Ca^2+^ transfer, lipid exchange, and synthesis, autophagy, and apoptosis (Rowland and Voeltz, 2012; Saheki and De Camili, 2017), thereby participating in the regulation of cardiovascular disease pathogenesis. However, the decrease of MAMs in senescent cells disrupts the physiological and pathological processes jointly regulated by the ER and mitochondria (Janikiewicz et al., 2018). For example, in aging cells, Ca^2+^ transport from ER to mitochondria is reduced (Fernandez-Sanz et al., 2014). Additionally, some studies have suggested that reducing age-related ER contacts may reduce the association with MAMs, thereby further triggering mitochondrial autophagy and age-related mitochondrial dysfunction (Palikaras et al., 2015). Therefore, this paper aims to elucidate the dual physical and functional connections between mitochondria and the ER and their interactions, further exploring the regulatory mechanisms for age-related CVDs to identify potential therapeutic targets.

## 2 Physical connection between mitochondria and endoplasmic reticulum: composition and structure of MAMs

MAMs are an important physical connection between mitochondria and the ER, crucial for maintaining cellular homeostasis, facilitating substance exchange, and regulating signal transduction for proper cellular function. The concept of MAMs dates back to 1969, first proposed by John Ruby and colleagues, and successfully isolated from rat liver tissue in 1990 (Vance, 1990). Subsequent advancements in techniques such as transmission electron microscopy, subcellular fractionation, and mass spectrometry, have provided a deeper understanding of the structural aspects of MAMs. MAMs comprise subregions of the ER and the outer mitochondria membrane (OMM), existing in close proximity without overlapping, typically maintaining a distance of 10–25 nm. However, this distance is dynamic, ranging from 10–100 nm under different conditions. For instance, it decreases during ER stress, hypoxia, and starvation (Bravo et al., 2011; Wu et al., 2016), while it increases under high-glucose environments (Theurey et al., 2016). Moreover, there are differences in distances between smooth ER and rough ER with mitochondria. High-resolution electron microscopy studies have indicated that the distance between the smooth ER and mitochondria at contact points is approximately 9–16 nm, while the distance between the rough ER and mitochondria falls within the range of 19–30 nm (Vance, 2014). MAMs can exhibit various structural morphologies within cells. For instance, ER tubules and mitochondria may align tangentially, with their contact surface constituting about 10% of the mitochondrial surface area. This type of MAM configuration is present in most cells. Additionally, ER tubules may fully or partially encircle mitochondria, covering approximately 50% of the mitochondrial circumference (Lan et al., 2019). To date, 1,000 different proteins have been identified within MAMs through in-depth mass spectrometry analysis and other techniques. These proteins can be categorized into three classes based on their localization within MAMs: MAMs-resident proteins, exclusively localized within MAMs; MAMs-enriched proteins, found in MAMs but also in other cellular compartments; and MAMs-associated proteins, transiently residing in MAMs under specific conditions (Poston et al., 2013).

The connection between ER subregions and the OMM is facilitated by a network of protein chains, serving as crucial bridges for stable and dynamic communication between these organelles. They are capable of interacting with regulatory proteins and are subject to their regulation. In yeast, this connection between the ER and mitochondria is mediated by the ER-mitochondria encounter structure (ERMES). ERMES is a complex composed of various proteins, including the ER-anchoring protein Mmm1, the cytoplasmic bridging protein Mdm12, as well as the OMM proteins Mdm34 and Mdm10, which regulate processes like phospholipid exchange, mitochondrial DNA replication, and maintenance of mitochondrial homeostasis, among others (Lang et al., 2015). In mammals, multiple protein complexes are implicated in linking the ER to mitochondria indeed more complex at MAMs sites:

(i) The IP3Rs-GRP75-VDAC Complex: This complex, pivotal in ER and mitochondria interplay, arises from the interaction between the Voltage-Dependent Anion Channel (VDAC) on the OMM and the 75-kDa glucose-regulated protein (GRP75), and inositol 1,4,5-triphosphate receptor (IP3R) on the ER. This complex regulates Ca^2+^ transport from the ER to the mitochondria, thereby modulating mitochondrial function and dynamics (Szabadkai et al., 2006). This process is regulated by α-Synuclein and glycogen synthase kinase-3 β (GSK-3 β) proteins. Sigma1R (Sig-1R) is involved in regulating cardiac function by interacting with other intimal reticulum partners via the IP3R-VDAC complex. It forms a Ca^2+^-sensitive complex by binding to Binding Ig Protein (BiP). The Sig-1R- BiP complex can effectively respond to changes in ER Ca^2+^ levels during signal transduction and affect Ca^2+^ transport between the ER and mitochondria by stabilizing IP3R (Reane et al., 2020). FUN14 domain-containing protein 1 (FUNDC1) is a highly conserved protein located on the OMM. It plays a crucial role in maintaining the formation of MAMs by interacting with IP3R2 and regulating Ca^2+^ transport and calcium homeostasis. The loss of FUNDC1 can result in a reduction in intracellular Ca^2+^ levels, leading to the inhibition of Ca^2+^-sensitive cAMP-response element binding protein (CREB), which subsequently downregulates the expression of Fis1. This can ultimately lead to mitochondrial dysfunction (Wu et al., 2017).
(ii) The VAPB-PTPIP51 Complex: This complex comprises vesicle-associated membrane protein-associated protein B (VAPB) and mitochondrial protein tyrosine phosphatase interacting protein 51 (PTPIP51). VAPB anchors to the ER membrane through its C-terminal transmembrane domain, while PTPIP51 is located in the OMM. Their interaction regulates calcium homeostasis and cellular autophagy. Disruption of the VAPB-PTPIP51 complex can lead to the uncoupling of ER-mitochondrial contacts and calcium imbalance (De Vos et al., 2012; Gomez-Suaga et al., 2017). Additionally, oxysterol-binding protein-related protein 5/8 (ORP5/8), found on the ER membrane, can establish a physical connection with PTPIP51. This interaction facilitates the transport of phosphatidylserine (PS) to the mitochondria. Inhibition of ORP5/ORP8 may potentially result in mitochondrial morphological defects (Galmes et al., 2016). Recent studies have also shown that the ER-anchored motile sperm domain-containing protein 2 (MOSPD2) interacts with PTPIP51, and both proteins play roles in cellular exchange and communication (Di Mattia et al., 2018).
(iii) Mitofusin 2 (MFN 2) in the ER assembles with MFN1/2 on the OMM to form homotypic or heterotypic dimers. MFN2, functioning as a GTPase, mediating mitochondrial fusion. Both the over-expression and knockdown of MFN2 can affect the interaction between the ER-mitochondria as well as Ca^2+^transfer, indicating that its role extends beyond mere physical connections through protein chains (Choi et al., 2006; Filadi et al., 2015).
(iv) BAP31-FIS1 Complex: This complex comprises mitochondrial fission one protein (Fis1) and B cell receptor-associated protein 31 (Bap31). Bap31, a partner protein located on the OMM, regulates the degradation of misfolded proteins and apoptosis pathways. The binding of Fis1 to Bap31 enables the transmission of apoptotic signals to the ER, thereby modulating the apoptosis pathway within the cell (Iwasawa et al., 2011). Additionally, the loss of the multifunctional sorting protein phosphofurin acidic cluster sorting 2 (PACS2) can lead to the generation of the pro-apoptotic fragment p20 Bap31, which subsequently modulates the interaction between the ER and mitochondria (Simmen et al., 2005).
(v) GRP78-WASF-ATAD3A Complex: This complex consists of the ER protein GRP78, the cytoplasm-localized protein WASF3, and the inner mitochondrial membrane (IMM) protein ATPase family AAA domain-containing 3A (ATAD3A). WASF3 traverses the OMM and binds to ATAD3A at its N-terminus. ATAD3A interacts with ER proteins such as MFN2, BiP, and dynamin-related protein 1 (Drp1) via WASF3 (Karbowski et al., 2002). These aforementioned protein complexes between the ER and mitochondria play a central role in maintaining the structural and functional integrity of MAMs.

## 3 Functional interactions between mitochondria and endoplasmic reticulum

### 3.1 Ca^2+^ transport

Ca^2+^, serving as crucial intracellular second messengers, plays pivotal roles in vital biological processes such as mitochondrial signaling and energy metabolism. Maintaining calcium homeostasis is indispensable for the functionality of key enzymes within the tricarboxylic acid cycle and respiratory chain complexes. The ER is considered the primary intracellular Ca^2+^ reservoir. Under physiological conditions, following the release of Ca^2+^ from the ER, it is transported to the mitochondrial matrix, where it participates in the tricarboxylic acid (TCA) cycle, thereby stimulating ATP synthesis. Nevertheless, a low concentration of Ca^2+^ in the mitochondria can result in disrupted energy metabolism, while an excessive concentration may lead to calcium overload, potentially triggering cellular apoptosis (Pinton et al., 2008). During the transfer of Ca^2+^ from the ER to the mitochondria, several proteins associated with the three layers of membranes, including the ER membrane, the OMM, and the mitochondrial inner membrane, are involved. Firstly, Ca^2+^ release is mediated by IP3Rs and Ryanodine Receptors (RyRs) located on the ER membrane. IP3Rs and RyRs serve as crucial Ca^2+^ efflux channels on the surface, creating high Ca^2+^ microdomains near the ER. Subsequently, Ca^2+^ enters the mitochondria through the VDAC located on the OMM. Grp75 bridges these two channels through its cytosolic domain, forming the IP3Rs-Grp75-VDAC complex (Szabadkai et al., 2006). Finally, Ca^2+^ enters the mitochondrial matrix through the Mitochondrial Calcium Uniporter (MCU) located on the mitochondrial inner membrane. However, the MCU exhibits a relatively low affinity for Ca^2+^. Research indicates that the formation of “high calcium microdomains” within the MAMs can stimulate MCU’s opening through elevated Ca^2+^ concentrations, allowing a rapid influx of Ca^2+^ into the mitochondria (De Stefani et al., 2016). Therefore, the presence of MAMs is crucial for Ca^2+^ transmission.

The Sarcoplasmic Reticulum (SR) is a membranous structure found in muscle cells such as cardiac and skeletal muscle cells. RyRs are predominantly expressed in the SR and are involved in the release of Ca^2+^ from the ER. They form a RyR2-VDAC2 complex with the VDAC, located on the mitochondrial outer membrane, mediating the transport of Ca^2+^ (Min et al., 2012). Recent research conducted by Jakob and Ryu et al. revealed that RyRs are partially located on the mitochondrial inner membrane. They identified the presence of RyRs on the mitochondrial inner membrane in both cardiac muscle cells (RyR1) and neuronal cells (RyR2), demonstrating their ability to cooperatively regulate mitochondrial calcium uptake along with the MCU (Ryu et al., 2010; Jakob et al., 2014). Additionally, Stoica et al. have discovered that PTPIP51 on the OMM can bind to the free end of VAPB located on the ER membrane. These two proteins form a complex that mediates the transport of Ca^2+^ (Stoica et al., 2014). Within the MAMs, Sig-1R is a Ca^2+^-sensitive receptor protein located on the ER that can form a complex with BiP. When the Ca^2+^ concentration in the ER decreases, this complex can detect changes in Ca^2+^ concentration and rapidly dissociate. It then binds to IP3Rs to stabilize their expression, thereby facilitating the continuous influx of Ca^2+^ into the mitochondria (Hayashi and Su, 2007). The Sig-1R- BiP complex efficiently responds to changes in ER Ca^2+^ levels and plays a crucial role in the ER-mitochondria Ca^2+^ signaling cascade. Lastly, recent research has revealed that the Transient Receptor Potential Cation Channel (TRPM8) functions as a functional ER Ca2+ release channel and plays a role in regulating mitochondrial Ca^2+^ homeostasis in VSMCs (Bidaux et al., 2018).

### 3.2 Mitochondrial dynamics

Mitochondria, highly dynamic organelles, undergo various processes including fusion, fission, and movement, resulting in diverse mitochondria forms moving along the cytoskeleton, forming fragmented structures or tubular mitochondrial networks (Nunnari and Suomalainen, 2012). Mitochondrial fusion contributes to the cell’s response to stress, while fission facilitates the removal of damaged or irreversibly dysfunctional mitochondria through mitophagy, inducing cell apoptosis. The protein Drp1 mediates mitochondrial fission, while MFN1/2 proteins, enriched in the MAMs, are involved in mitochondrial fusion (de Brito and Scorrano, 2008; Saotome et al., 2008). Hence, MAMs play a critical role in these processes.

During mitochondrial fission, the tubular ER membrane approaches mitochondria and wraps around its constriction site. Subsequently, Drp1 is recruited to the OMM, where it interacts with adapter proteins such as FIS1, MFF, MiD49, and MiD51, facilitating mitochondria division. Drp1 activity is regulated by phosphorylation modifications mediated by Protein Kinase A (PKA) or Ca^2+^/calmodulin-dependent protein kinase Iα (CaMKIα) (Chang and Blackstone, 2007; Han et al., 2008). Additionally, redox signaling regulates Drp1 activity, as increased mitochondrial reactive oxygen species (ROS) can promote mitochondrial fission by oxidizing Drp1 (Navaneetha Krishnan et al., 2020). Several other MAM-associated molecules are involved in mitochondrial fission. For instance, Inverted Formin 2 (INF2) promotes actin polymerization, aiding mitochondrial constriction, and relocating Drp1 from the cytoplasm to the mitochondrial division site to initiate fission (Steffen and Koehler, 2018). Moreover, the knockout of MCU located on MAMs reduces mitochondrial fission (Chakrabarti et al., 2018).

Mitochondrial fusion involves the fusion of the IMM and the OMM, with core molecules including MFN1/2 and optic atrophy 1 (OPA1) (Chan, 2006). MFN1 is localized to mitochondria, MFN2 is present not only on the mitochondrial outer membrane but also enriched in the ER membrane and MAMs. MFN2 connects the ER to mitochondria and stabilizes MAM formation. OPA1 cooperates with MFN1 to mediate fusion of the mitochondrial inner membrane, while MFN2 connects both organelles by forming homotypic or heterotypic dimers with MFN1 or MFN2 on the mitochondrial surface, regulating outer mitochondrial membrane fusion and mitochondrial calcium uptake (de Brito and Scorrano, 2008). Recent research has identified that FUNDC1, located on the OMM, can interact with the IMM protein OPA1, facilitating mitochondrial fusion (Chen et al., 2016).

Mitochondrial movement is primarily regulated by the Mitochondrial Rho GTPase 1 (Miro1) and Miro2 protein families, which interact with motor proteins to mediate mitochondrial movement along the cellular cytoskeleton. Since high Ca^2+^ concentrations are required for mitochondrial movement along the cytoskeleton, and Miro1 and Miro2 have low Ca^2+^ affinities, MAMs also play a significant role in this process (Wang et al., 2011).

### 3.3 Lipid synthesis and transport

Lipids are a general term for triglycerides (TG) and lipoids, which include cholesterol and its esters, phospholipids, and glycolipids. Serving as an important component of biofilm, lipids play a variety of important biological functions in cell biology, including energy storage, regulation of cell conduction, and synaptic transmission, as well as constituting various physiological active substances. The ER serves as the primary site for lipid synthesis within cells. The hydrophobic lipids synthesized in the ER are primarily transported through vesicular transport mechanisms within the aqueous cytoplasm to other cellular organelles. However, recent research indicates that lipid transfer between mitochondria and the ER occurs independently of vesicular transport, relying instead on interactions at membrane contact sites known as MAMs (Stone et al., 2009). Moreover, during the lipid synthesis process within the ER, crucial enzymes on the membranes of organelles such as mitochondria also play a pivotal role. Mitochondria, in particular, assume a critical role in the synthesis of steroidal hormones, a process requiring the transport of intracellular free cholesterol across the mitochondrial inner membrane, where it serves as a substrate in the reactions. Recent research has revealed that the transmembrane transport of cholesterol from the IMM to the OMM represents the rate-limiting step in the synthesis of steroidal hormones (Miller, 2007; Prasad et al., 2015). This transmembrane process involves the participation of various proteins, including the steroidogenic acute regulatory protein (StAR), which facilitates the transport of cholesterol from the cytoplasm to the mitochondrial inner membrane. The StAR protein can combine with proteins associated with the mitochondrial membrane, facilitating the formation of hydrophobic channels on the OMM. Notably, Papadopoulos and colleagues discovered that only properly folded StAR can exhibit its biological activity (Papadopoulos et al., 2015). Further investigations into the regulatory mechanisms governing StAR folding have revealed that the VDAC and Translocator Proteins (TSPO) located on MAMs can interact with StAR, participating in its folding process. Furthermore, StAR, VDAC1, TSPO, and Acyl-CoA Binding Domain Containing 3 (ACBD3) can form complexes that play a role in cholesterol transport (Liu et al., 2006).

The most abundant class of phospholipids found within cells is phosphatidylethanolamine (PE), often referred to as “brain phospholipid.” In mammalian cells, PE synthesis primarily occurs through the “PS decarboxylation pathway,” a pivotal process predominantly taking place within the MAMs. Several proteins likely participate in the synthesis and transport of phospholipids within MAMs. Phosphatidylserine synthase 1/2 (PSS1/2) catalyzes the conversion of phosphatidic acid (PA) into PS. Subsequently, PS is transferred to the IMM, with research suggesting that the transmembrane transport of PS is mediated by the ORP5/8-PTPIP51 protein complex enriched within MAMs (Sohn et al., 2018). Within the IMM, PS is further converted into PE by phosphatidylserine decarboxylase (PSD). The synthesized PE is then transported out of the mitochondria and, under the catalysis of phosphatidylethanolamine N-methyltransferase 2 (PEMT2), is converted into phosphatidylcholine (PC) (Krols et al., 2016). The ratio of PC to PE closely correlates with cellular membrane integrity, with the transmembrane transport of PS from the ER to the mitochondria being considered a rate-limiting step in PE synthesis. This underscores the significant role of MAMs in maintaining cellular membrane integrity.

The diglyceride pathway represents one of the crucial routes for TG synthesis, with Diacylglycerol Acyltransferase 2 (DGAT2) playing a pivotal role as a catalytic enzyme. DGAT2 efficiently catalyzes the conversion of diacylglycerols (DAGs) into triglycerides (TAGs), thus serving as the principal rate-limiting enzyme in this pathway. Research findings have shown that mice with a knocked-out DGAT2 gene die shortly after birth due to TG deficiency (Stone et al., 2004). Scot et al.'s study confirmed the dynamic distribution of DGAT2 on the ER, MAMs, and mitochondria through techniques such as immunofluorescence and biochemical fractionation. Notably, DGAT2 exhibited its highest activity within the MAMs (Stone et al., 2009). Long-chain fatty acid coenzyme A (CoA) ligase 4 (FACL4) serves as a vital marker within MAMs and plays a pivotal role in TG synthesis and transport. It can convert acetyl-CoA, derived from sources such as glucose, into acyl-CoA, subsequently leading to the generation of diacylglycerol (DAG) and triglycerides (TAG). Additionally, tether proteins within MAMs, such as cholesterol acyltransferase/sterol O-acyltransferase 1 (ACAT1/SOAT1), are also crucial participants and platforms in lipid biosynthesis and transport processes (Anastasia et al., 2021).

### 3.4 Autophagy

Autophagy is an intracellular degradation pathway unique to eukaryotic organisms, reliant on lysosomes. It plays a crucial role in maintaining cellular tissue homeostasis and exhibits a high degree of selectivity. Under physiological conditions, autophagy serves as a cellular self-defense mechanism, selectively removing misfolded proteins and damaged organelles, thereby protecting cells from oxidative and metabolic stress damage. It is an integral component of the cellular protein quality control system (Sciarretta et al., 2018; Zhang et al., 2018). However, excessive autophagy can lead to the over-degradation of cellular components, including proteins and organelles, and may even trigger cell death. The process of autophagy involves several key stages. Firstly, the formation of the phagophore with a double-layer membrane structure occurs. Subsequently, the phagophore extends continuously to encapsulate cell elements or autophagy-related molecules, forming a closed spherical autophagosome. Finally, the autophagosome fuses with lysosomes, leading to the degradation and recovery of substrates (Aman et al., 2021). These stages of autophagy are tightly regulated by a series of molecules, including MAM proteins.

The process of autophagy begins in a region rich in phosphatidylinositol-3-phosphate (PI3P) at the ER-mitochondrial coupling site. ATG14, a marker of autophagy precursor, is involved in the formation of autophagosomes (Diao et al., 2015). It forms a PI3 kinase complex with VPS34, p150, BECN1, and NRBF2. This complex, along with the unc-51-like autophagy activating kinase 1 (ULK1) complex, mediates the extension of phagophores during autophagy. Under resting conditions, ATG14 is primarily localized to the cytoplasm and the ER. However, under starvation conditions, the localization of ATG14, along with the autophagy-associated protein double FYVE domain-containing protein 1 (DFCP1), shifts notably to the MAM region. Similarly, the ATG conjugation system is involved in the process of phagophores eventually maturing and sealing to form autophagosomes. ATG5 relocates from other cellular locations to the MAM region during this process, subsequently detaching from MAMs after autophagosome maturation (Hamasaki et al., 2013). Further research has revealed that disturbance in ER-mitochondria tethering proteins (such as PACS2 and MFN2) impairs the precise localization of ATG14 and DFCP1 to MAMs, leading to a significant inhibition of autophagosome formation. This underscores the critical regulatory role of MAM proteins in the autophagy process. Furthermore, other key molecules involved in autophagy, such as PTEN-induced putative kinase1 (PINK1)/Parkin and BECN1, also localize to MAMs. PINK1 possessing serine/threonine kinase activity, collaborates with the E3 ubiquitin ligase Parkin to mark and process damaged mitochondria through the autophagy pathway. Additionally, PINK1 can directly interact with BECN1, promoting contact between mitochondria and the ER, as well as the formation of autophagosome precursor structures (omegasomes). Silencing PINK1 results in a reduced enrichment of BECN1 on MAMs, consequently inhibiting the autophagic process (Gelmetti et al., 2017; Barazzuol et al., 2020).

In addition to regulating autophagy-related proteins, MAMs also modulate the autophagic process by controlling the synthesis and transport of lipids and Ca2+. Phospholipids and sterols in lipids play significant roles in autophagosome formation. During the extension of the phagophore, PE can bind to the autophagy-related protein LC3, promoting the fusion and closure of LC3-mediated phagophores (Nair et al., 2012). Additionally, lipid droplets (LDs), cellular organelles for storing lipids, serve as essential sources of lipids required for autophagosome synthesis. Ca^2+^ imbalance in MAMs can also lead to autophagic abnormalities. The VAPB-PTPIP51 complex regulates autophagy by mediating Ca^2+^ transfer from the ER to the mitochondria, where it negatively regulates mitochondrial autophagy. Disrupting Ca^2+^ transport mediated by IP3Rs can affect this regulatory function (Gomez-Suaga et al., 2017).

### 3.5 Endoplasmic reticulum stress

The ER serves as the primary Ca^2+^ storage site within cells and functions as the major processing center for protein synthesis, folding, and transport. With the assistance of chaperone proteins, the ER achieves optimal protein folding while preventing protein aggregation under normal physiological conditions. However, under various stresses, unfolded and misfolded proteins can accumulate, disrupting ER homeostasis and triggering the unfolded protein response (UPR). The UPR alleviates excessive protein load through mechanisms such as expanding the ER membrane and reducing protein synthesis (Hetz, 2012). Moderate UPR aids in cell survival, but sustained ER stress may lead to apoptosis. During the UPR, three parallel signaling pathways are activated: protein kinase-like ER kinase (PERK), inositol-requiring enzyme 1 (IRE1), and activating transcription factor 6 (ATF6). The PERK pathway inhibits protein translation by phosphorylating eIF2α, reducing protein influx into the ER. The IRE1α pathway achieves a similar effect by degrading mRNA transcripts encoding certain ER proteins through regulated IRE1-dependent decay (RIDD) (Han et al., 2009). Unlike PERK and IRE1α, ATF6 increases the folding and degradation capacity of ER proteins to alleviate ER protein load. Under normal physiological conditions, PERK, IRE1α, and ATF6 are bound to Binding Immunoglobulin Protein (BiP) and exist in an inactive form in the ER-lumenal domains. However, during ER stress, a large accumulation of unfolded proteins binds to BiP, leading to the release of PERK, IRE1α, and ATF6α, initiating the UPR response (Yuan et al., 2020).

The UPR process is regulated by various MAMs-tethering proteins. Research has demonstrated that ER stress signals are modulated by MFN2 expressed on MAMs. MFN2 knock out can induce ER stress and enhance the activity of all three UPR pathways, while simultaneously reducing autophagy and apoptosis associated with ERS. Furthermore, MFN2 can physically interact with the PERK protein, acting as an upstream regulator of PERK activity (Ngoh et al., 2012). Another tethering protein in MAMs, VAPB, can directly interact with ATF6, reducing ATF6 activity. Furthermore, the P56S mutation in VAPB can disrupt the IRE1α/XBP1 pathway (Tokutake et al., 2015). Other proteins that disrupt ER-mitochondria connections and communication can also activate the UPR process, such as Sig1R, cyclophilin D (CypD), and PACS2 (Simmen et al., 2005; Rieusset et al., 2016). Moreover, several UPR-related proteins are enriched in the MAMs and contribute to these cellular processes. For instance, the presence of IRE1α in MAMs can enhance cell survival through Xbp1 mRNA splicing, while promoting apoptosis via mitochondrial calcium overload (Shuda et al., 2003). Moreover, the ubiquitination of IRE1α might inhibit UPR-induced cell apoptosis. Similarly, PERK is enriched in MAMs, promoting ER-mitochondria interaction and promoting ROS-triggered mitochondrial-mediated cell apoptosis (Verfaillie et al., 2012). These research findings underscore the crucial regulatory role of MAMs in the ER stress process.

### 3.6 Other

In addition to their role in regulating the aforementioned cellular physiological and pathological activities, MAMs are closely involved in cellular processes such as inflammation and apoptosis. They exert influence on these processes by recruiting regulatory factors and transmitting signals through various pathways. The inflammasome, a multiprotein complex is crucial in inflammatory signaling. MAMs are intricately linked with the assembly and activation of the NOD-, LRR-, and pyrin domain-containing protein 3 (NLRP3) inflammasome, thereby regulating the inflammatory response. In its inactive state, NLRP3 is distributed within the ER and cytoplasm. Upon stimulation by various danger signals, NLRP3, along with the apoptosis-associated speck-like protein containing a CARD (ASC), is recruited to MAMs, facilitating the activation of the NLRP3 inflammasome, subsequently activating caspase-1. Activated caspase-1 catalyzes the maturation and secretion of pro-interleukin-1β (pro-IL-1β) and pro-interleukin-18 (pro-IL-18), triggering an inflammatory response and caspase-1-dependent cell death (Zhou et al., 2011).

Mitochondria are intimately linked with cell apoptosis, and MAMs play a crucial role in regulating apoptosis by modulating mitochondrial morphology, structure, and function. In the intrinsic cellular apoptosis pathway involving mitochondria, the MAMs-mediated transfer of Ca^2+^ from the ER to mitochondria is considered a critical event. Mitochondrial calcium overload can induce the opening of mitochondrial permeability transition pores (mPTP), releasing pro-apoptotic factors such as cytochrome c and apoptosis-inducing factor (AIF), ultimately triggering cell apoptosis (Zhang et al., 2016). The FIS1-BAP31 complex within MAMs transmits mitochondrial apoptosis signals to the ER by recruiting and activating caspase-8, thereby regulating cell apoptosis (Iwasawa et al., 2011). Additionally, MAM complexes involved in Ca^2+^ transport, such as IP3Rs-GRP75-VDAC1, play a significant role in regulating cell apoptosis. Inhibiting the expression of IP3R has been shown to effectively decrease cell apoptosis induced by both extrinsic and intrinsic apoptotic pathways (Mendes et al., 2005). Furthermore, the mitochondrial fusion complex MFN2-MFN1/2 can influence apoptotic pathways by regulating the stability of MAMs.

**FIGURE 1:**
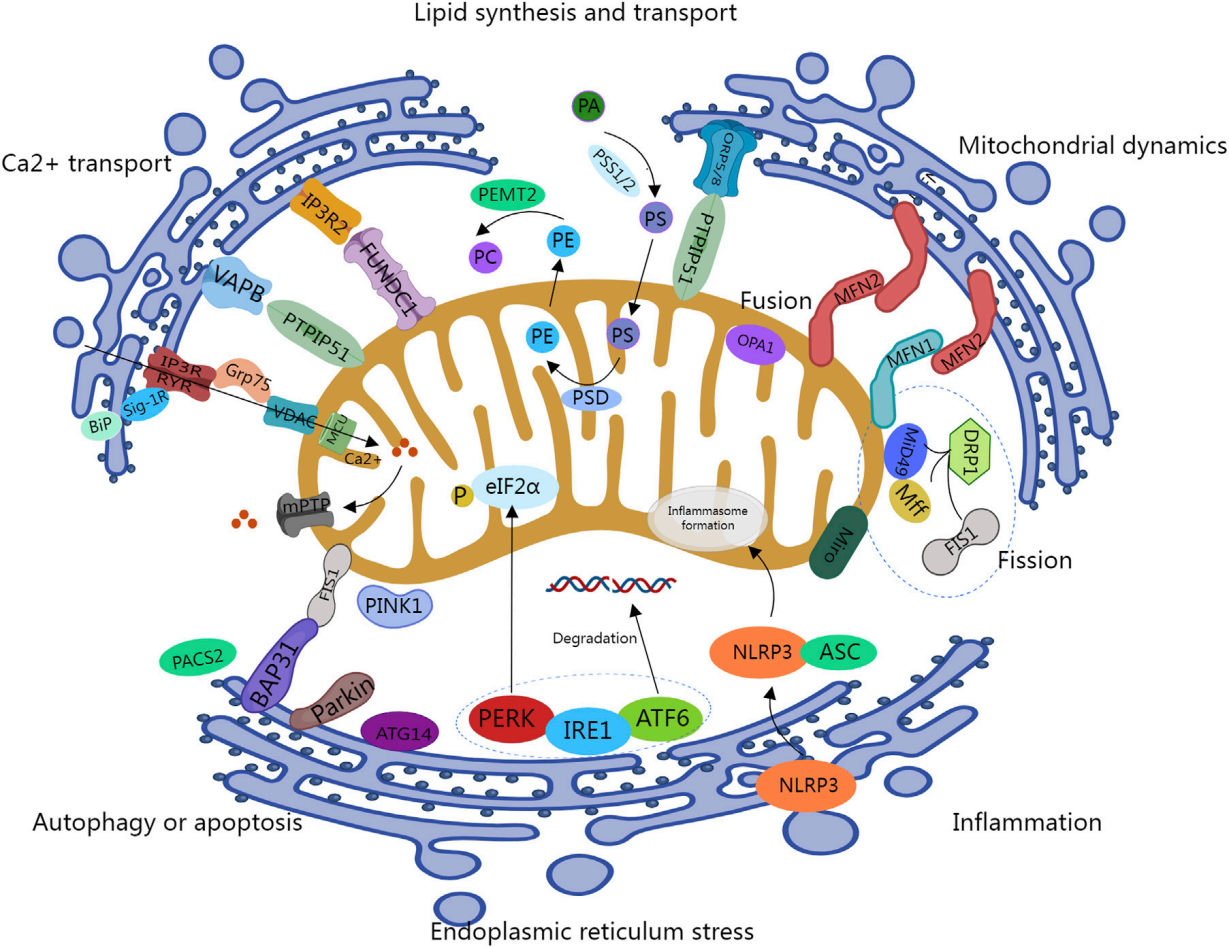
Key cellular functions handled at the Mitochondrial-associated ER Sites. IP3R, inositol 1,4,5-triphosphate receptor; GRP75: 75-kDa glucose-regulated protein; VDAC, Voltage-Dependent Anion Channel; BiP, Binding Ig Protein; Sigma1R, Sig-1R; MCU, Mitochondrial Calcium Uniporter; mPTP, mitochondrial permeability transition pores; VAPB, vesicle-associated membrane protein-associated protein B; PTPIP51, protein tyrosine phosphatase interacting protein 51; FUNDC1, FUN14 domain-containing protein 1; ORP5/8, oxysterol-binding protein-related protein 5/8; PSS1/2, phosphatidylserine synthase 1/2; PSD, phosphatidylserine decarboxylase; PEMT2, phosphatidylethanolamine N-methyltransferase 2; MFN1/2, Mitofusin 1/2; OPA1, optic atrophy 1; DRP1, dynamin-related protein 1; Fis1, fission one protein; Miro, Mitochondrial Rho GTPase; PACS2, phosphofurin acidic cluster sorting 2; Bap31, B cell receptor-associated protein 31; PINK1, PTEN-induced putative kinase1; PERK, protein kinase-like ER kinase; IRE1, inositol-requiring enzyme 1; ATF6, activating transcription factor 6; NLRP3, NOD-, LRR-, and pyrin domain-containing protein 3; ASC, apoptosis-associated speck-like protein containing a CARD.

The above-mentioned functions of MAMs in cell physiology are depicted in Figure 1.

## 4 Pathophysiologic changes associated with the aging process

### 4.1 Relevance of cardiovascular system alterations to aging

Aging is closely linked with the gradual decline of numerous physiological processes and is widely recognized as a significant risk factor for CVDs. The aging process significantly affects the structure and function of the heart and arterial system, leading to pathological changes such as myocardial hypertrophy, left ventricular functional alterations, vascular endothelial injury, and increased arterial stiffness (Lakatta and Levy, 2003). These changes can ultimately lead to the development of CVDs, including atherosclerosis, myocardial infarction, arrhythmias, and heart failure.

The structural and functional changes in arteries during aging increase the risk of CVDs. Key vascular changes primarily include systemic endothelial dysfunction and central arterial stiffening. Endothelial dysfunction, closely associated with vascular aging, is characterized by reduced vasodilation, impaired anti-thrombotic ability, increased inflammatory factors, and oxidative stress, among other factors (Donato et al., 2007; Delp et al., 2008). These represent early steps in atherosclerosis development. Research indicates that (aging can reduce the expression of endothelium-derived nitric oxide synthase (eNOS), thereby decreasing the synthesis and bioavailability of nitric oxide (NO), a crucial factor in combating atherosclerosis and the basis of age-related endothelial dysfunction (Lüscher et al., 1989). Additionally, aging may lead to the accumulation of ROS due to inadequate superoxide dismutase activity in ECs, along with disturbances in lipid metabolism resulting from altered expression of cholesterol transporters (Wong et al., 2019). Furthermore, aging vascular ECs may exhibit increased expression of adhesion molecules while levels of thrombomodulin decrease. Collectively, these factors contribute to atherosclerosis development. Lakatta and Levy. (2003) observed that the aging heart typically experiences a characteristic structural change marked by an increase in the mass-to-volume ratio, accompanied by a notable reduction in left ventricular end-diastolic volume. Thickening of the interventricular septum and thinning of the ventricular free wall, exhibiting asymmetric changes, may underlie this phenomenon. Early stages may see cardiac myocyte hypertrophy, progressing to compensatory fibrosis due to impaired cardiac function, leading to arrhythmias and degenerative changes in the myocardium, ultimately leading to heart failure. Elderly patients are often more susceptible to heart failure with preserved ejection fraction (HFpEF), a condition closely associated with arterial stiffness, myocardial extracellular matrix, and microvascular remodeling, with the risk increasing significantly with age (Lloyd-Jones et al., 2002).

Furthermore, the pathological processes of many CVDs are closely related to aging. For example, as individuals age, calcium accumulation in cardiovascular structures may lead to aortic valve calcification. Evidence suggests that inflammation may contribute to cardiovascular calcification, with research indicating a significant role for lipoprotein(a) in this pathological process (Thanassoulis et al., 2013). Another notable factor associated with the elderly population is certain forms of amyloidosis. Novel imaging techniques have revealed that about 13% of HFpEF patients over 60 years old exhibit wild-type transthyretin (wtTTR) amyloidosis (González-López et al., 2015). Further research has revealed that in the population aged 80 and above, at least 20% of hearts exhibit deposits of wtTTR amyloid protein. While the clinical significance of this amyloidosis is not entirely understood at present, it holds promise for novel approaches to diagnosing and treating heart failure in the elderly population.

### 4.2 Relevance of mitochondrial, endoplasmic reticulum and its membrane sites MAMs to aging

Aging is often accompanied by a series of changes in cell function, which is closely related to the age-related changes of organelles in cells. Up to now, degeneration of mitochondrial function has been regarded as an important sign of aging and one of the main factors contributing to aging and age-related disease manifestations (Javadov et al., 2020), closely associated with degenerative changes in the nervous system, cognitive functions, CVDs, etc. Animal studies have revealed aging-related changes in mitochondrial morphology and bioenergetics, including mitochondrial damage, cristae loss, or disorder loss (Lyu et al., 2020). Mitochondrial membrane potential (mtΔΨ) plays a crucial role in the degradation of damaged mitochondria and cell survival and is closely associated with mitochondrial dysfunction. Its level changes with age, with studies indicating a significant decrease in mtΔΨ in the liver cells of elderly rats compared to young rats (Kwong and Sohal, 2000). Furthermore, recent research has implicated another important feature of mitochondrial dysfunction, mitochondrial permeability transition (MPT), in the mechanisms of aging (Panel et al., 2018). While there is no substantial evidence suggesting significant changes in the physiological function of the ER during aging, Druelle et al. observed an expansion of the ER in normal human fibroblasts (NHFs) during the aging process using selective fluorescent probes (Druelle et al., 2016). In certain diseases such as heart disease, changes in the ER environment, including calcium levels, molecular chaperones, and oxidative stress, may lead to the accumulation of misfolded proteins and protein imbalance, triggering the UPR. Elevated UPR has been observed in aging fibroblasts.

**FIGURE 2:**
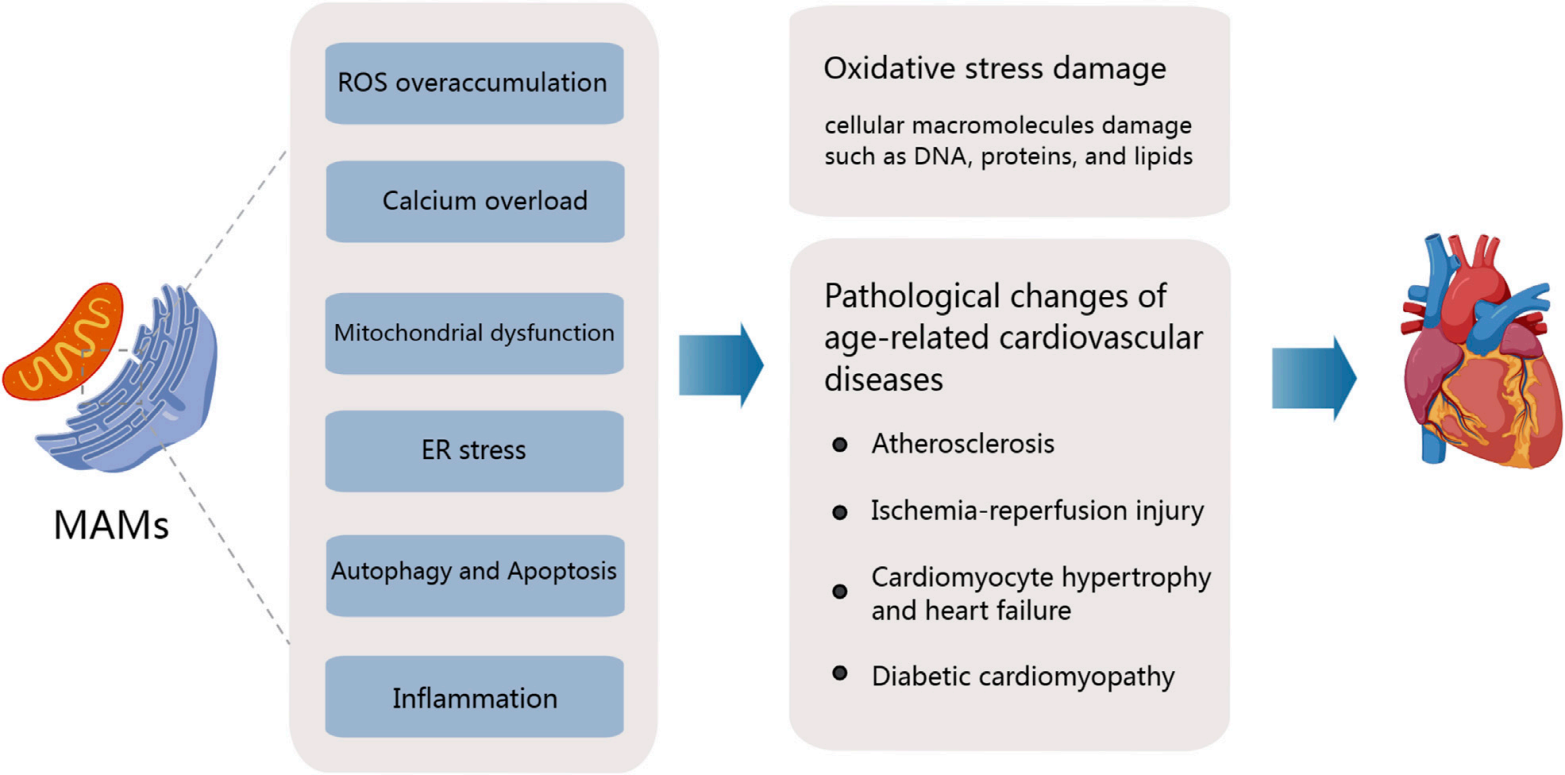
Mitochondria-associated ER membranes and pathogenesis of age-related cardiovascular diseases. The aging process significantly affects the structure and function of the heart and arterial system, accompanied by the degradation of mitochondrial function and the overproduction of ROS. Major MAM-mediated abnormalities leading to age-related CVD include ROS overaccumulation, calcium overload, Mitochondrial dysfunction, Er stress, the activation of inflammation response, and the disorders of apoptosis and autophagy.

Different organelles interact to facilitate the transfer of substances and communication between each other. These are called membrane sites. (Wong et al., 2019). Among them, MAMs, as an important connection between mitochondria and endoplasmic reticulum, has been implicated in the regulation of various cellular functions, including calcium transport, mitochondrial dynamics, autophagy, lipid synthesis and transport, and oxidative stress. These processes are affected during the early stages of aging (Müller et al., 2018), with a decrease in the number of MAMs observed in aging cells. In a Huntington’s disease mouse model, Cherubini et al. observed significant decreases in the levels of proteins such as IP3R, Grp75, and MFN2 in MAMs with age, suggesting that changes in the content of these proteins could disrupt ER and mitochondrial connections during aging (Cherubini et al., 2020). In another study, Madreiter-Sokolowski et al. demonstrated that enhancing tethering between the ER and mitochondria in aging ECs can increase Ca^2+^ levels, although these cells are more susceptible to cell death due to calcium overload (Madreiter-Sokolowski et al., 2019). These insights offer a preliminary understanding of the changes in MAMs during aging. In the subsequent sections, we further discuss their role in regulating the pathological and physiological mechanisms of age-related CVDs (Figure 2).

## 5 MAMs mediate oxidative stress responses during aging contributing to age-related cardiovascular disease

Harman’s free radicals theory of aging (FRTA) is one of the main theories proposed to explain the mechanism of aging. Harman believes that aging is characterized by a decline in the body’s antioxidative capacity, particularly in the ability to eliminate oxygen free radicals, leading to the accumulation of endogenous free radicals such as ROS, which further induces oxidative damage of cellular macromolecules such as DNA proteins and lipids (HARMAN, 1956; Sies and Cadnas, 1985). Aging-related oxidative stress significantly elevates the risk of CVDs such as hypertension, atherosclerosis, myocardial infarction, and heart failure. This oxidative stress is considered a major contributor to the pathogenesis of age-related CVDs. The regulation of ROS plays a crucial role in various aspects of CVDs, which we will now explore: (i) Hypertension: Within the cardiovascular system, the primary sources of ROS include nicotinamide adenine dinucleotide phosphate (NADPH) oxidase (NOX), eNOS, and xanthine oxidase (XO). Among these, the activation of NOX and the uncoupling of eNOS play pivotal roles in the regulation of hypertension (Matsuno et al., 2005). Angiotensin II, for example, can elevate blood pressure by activating vascular NOX and ROS originating from NOX. (ii) Atherosclerosis: Low-density lipoprotein (LDL) functions as the primary carrier of cholesterol in the body, and the oxidation of LDL is a major event in the early stages of atherosclerosis. ROS can oxidize LDL, converting it into oxidized LDL (OX-LDL), which accumulates within the endothelium and promotes the formation of atherosclerotic plaques. (iii) Ischemia-reperfusion injury: During cardiovascular events such as ischemia-reperfusion injury (IRI), a significant amount of ROS is generated, potentially leading to myocardial infarction (Braunersreuther and Jaquet, 2012). Studies have identified elevated levels of NOX2 protein in the myocardial cells of individuals who have died due to acute myocardial infarction. (iv) Vascular dysfunction: ROS also can contribute to severe vascular dysfunction by reducing the levels of the antioxidant stress factor Nrf2, decreasing the bioavailability of NO, and increasing the production of vasoconstrictive molecules (Zhang and Gutterman, 2007).

Mitochondrial, ER and its membrane sites MAMs, participate in the regulation of ROS production and contribute to the pathogenesis of CVDs. ROS are generated through the redox reactions of three cellular organelles: mitochondria, peroxisomes, and the ER, collectively referred to as the " REDOX triangle” (Yoboue et al., 2018). During oxidative metabolism and ATP synthesis, the mitochondrial electron transport chain (ETC.) serves as the primary source of ROS generation within the mitochondria. Studies have demonstrated that excessive Ca^2+^ transfer from the ER to the mitochondrial cristae, mediated by MAMs, can promote ROS production and lead to the formation of redox nanodomains at the MAMs interface (Booth et al., 2016). During this process, the accumulation of Ca^2+^ within the mitochondria can cause mitochondrial depolarization and aberrant oxidative phosphorylation, facilitating ROS generation by uncoupling the mitochondrial electron transport chain from respiratory Complexes I and III (Wu et al., 2017). ROS, in turn, can enhance Ca2+ influx into the mitochondrial matrix by oxidizing the MCU.

While the ER has a smaller impact on oxidative stress compared to mitochondria, it remains an important source of ROS. Within the ER, ROS are primarily generated by cytochrome P450 family proteins, NADPH oxidase 4 (Nox4), and ER oxidoreductin (Ero1) (Janikiewicz et al., 2018). For example, Ero1α can induce the oxidation of IP3R1, leading to the dissociation of IP3R1 from ER-resident protein 44 (ERp44), thereby enhancing Ca^2+^ transport flux and resulting in ROS production.

Furthermore, many structural proteins of MAMs are involved in the process of ROS production. For instance, the 66-kDa Shc isoform (p66Shc), located in the MAMs, undergoes phosphorylation at the Ser36 residue by the oxidatively activated protein kinase Cβ (PKCβ), subsequently entering the mitochondria to induce ROS production (Bhat et al., 2015). These findings highlight the significant role of MAMs in oxidative stress, ROS generation, and signal transduction processes, further underscoring their crucial regulatory role in age-related CVDs.

## 6 MAMs in regulating pathological mechanisms of age-related cardiovascular diseases

### 6.1 Vascular endothelial damage and atherosclerosis

Atherosclerosis, a complex cardiovascular disease, results from multiple factors, including lipid metabolism imbalances, vascular endothelial dysfunction, and chronic inflammation. It significantly contributes to the increasing incidence and mortality rates of CVDs over the years. In the arterial intima, the accumulation of LDL represents the initial step in atherosclerosis development (Ference et al., 2017). Elevated LDL levels facilitate its penetration and retention within the subendothelial space of arteries. Subsequently, oxidized OX-LDL, produced after oxidation, stimulates ECs to secrete chemotactic factors, further activating the inflammatory response. Immune cells, primarily monocytes, infiltrate the arterial intima and differentiate into macrophages, which engulf OX-LDL and transform into lipid-laden foam cells (Brown and Goldstein, 1983), thus initiating atherosclerosis. These activated macrophages also secrete pro-inflammatory cytokines, exacerbating the inflammatory response (Chinetti-Gbaguidi et al., 2015), thus further aggravating AS.

MAMs play a significant role in the pathological processes associated with AS, including endothelial dysfunction, VSMCs changes, lipid infiltration, and inflammatory responses, among others. Endothelial dysfunction is an early event in the occurrence and development of AS. Under physiological conditions, ECs protect arteries from AS and reduce inflammation. However, apoptosis induced by OX-LDL becomes the initiation step of AS when EC is damaged. Excessive OX-LDL accumulation can lead to elevated mitochondrial Ca^2+^ and ROS levels, loss of mitochondrial membrane potential (MMP), and cytochrome c release, thereby inducing ECs apoptosis, which is related to mitochondrial calcium overload (Yu et al., 2019). PACS2, as an ER-mitochondrial tethering protein, participates in the occurrence of calcium overload by regulating Ca^2+^ transfer in MAMs. Relevant studies have demonstrated that silencing PACS2 in OX-LDL-induced apoptosis model of human umbilical vein endothelial cells (HUVECs) can inhibit OX-LDL-induced EC cell apoptosis, MAMs formation, and Ca^2+^ increase. In addition, some studies have reported that disruption of MAMs in ECs can mitigate mitochondrial damage, apoptosis, and inflammatory responses while increasing NO release (Yang et al., 2019).

Aging, inflammation, oxidative stress, and other factors can induce stressful changes in VSMCs, contributing to the development of AS. Numerous studies have established the critical role of VSMCs in regulating the structure of atherosclerotic plaques, specifically the fibrous cap and necrotic core. In VSMCs, saturated lysophosphatidic acids (LPAs) accumulate at contact sites between omegasomes and MAMs through activation of glycerol-3-phosphate acyltransferase 4 (GPAT4). This accumulation disrupts the autophagy flux, leading to the development of severe vascular calcifications (Shiozaki et al., 2020). These calcifications contribute to atherosclerotic plaque instability. Interestingly, a separate study by Moulis et al. suggests that MAMs play a critical role in regulating the balance between cell survival and death in VSMC apoptosis models (Moulis et al., 2019). This function is achieved by modulating the interplay between autophagy and apoptosis. Moulis et al. reported for the first time that under the influence of OX-LDL, the expression of PACS-2 in the MAMs region of VSMCs increases. Silencing PACS-2 disrupts MAM structure, impairing mitochondrial autophagy, and promoting VSMC apoptosis. This suggests that MAMs may serve as a novel target for improving VSMC and stabilizing atherosclerotic plaque.

Furthermore, AS is considered chronic inflammation of the vascular wall, so MAMs, as an important platform of the inflammatory response, play an important role in this process. Aging is indeed a significant risk factor for atherosclerosis, evidenced by cellular aging within AS plaque lesions, characterized by reduced cell proliferation, apoptosis, DNA damage, epigenetic modifications, and functional impairments (Wang and Bennett, 2012; Head et al., 2017). Increasing evidence suggests that cellular aging promotes AS onset and progression.

### 6.2 Ischemia-reperfusion injury

Ischemia-reperfusion injury refers to the pathological phenomenon where cellular or tissue structural damage and functional impairment worsen progressively following the restoration of normal blood flow to the ischemic myocardium. It is primarily associated with excessive ROS generation within myocardial cells, mitochondrial dysfunction, calcium overload, ER stress, and disruptions in energy metabolism (Frank et al., 2012; Heusch, 2020). Meanwhile, the body aging process is accompanied by increased oxidative stress damage, alterations in aging-related signaling molecules and other stress factors. Due to the reduced self-repair capability of aging cells, this may further contribute to cardiac dysfunction and adverse effects on stress responses. Studies suggest that calcium overload, a consequence of disrupted calcium homeostasis, is a critical mediator of IRI. This overload is likely achieved through the opening of the mPTP (mitochondrial permeability transition pore). This pathological process may trigger the release of pro-apoptotic factors, thereby inducing cell apoptosis. (Paillard et al., 2013; Zhang et al., 2016). Studies have confirmed that following IRI, protein complexes such as IP3Rs-Grp75-VDAC in MAMs mediate the transfer of Ca^2+^ from the ER to the mitochondria, leading to calcium overload. Excessive accumulation of Ca^2+^ in the mitochondria can activate CypD located in the mitochondrial matrix, making it an important regulatory factor for mPTP opening (Elrod and Molkentin, 2013). Moreover, Paillard et al. demonstrated that CypD can interact with IP3Rs-Grp75-VDAC during the process of Ca^2+^ transfer from the ER to the mitochondria (Paillard et al., 2013). By silencing the Ppif gene encoding CypD, they discovered that mice lacking CypD were able to prevent IRI-induced cell death (Baines et al., 2005). Glycogen synthase kinase-3 beta (GSK-3β), as a regulatory factor for Ca^2+^, can also interact with IP3 complexes, participating in the transport of Ca^2+^ within myocardial cells. By inhibiting GSK-3β, it is possible to mitigate calcium overload in IRI, thereby reducing myocardial cell apoptosis (Gomez et al., 2015). Moreover, the decrease in MFN2 expression may alleviate mitochondrial calcium overload and cellular damage by preventing the interaction between CypD and VDAC1-IP3R1. This underscores the pivotal role of MAMs in regulating mPTP and Ca^2+^ channels, making them promising therapeutic targets for IRI management. Mitochondrial dynamics also play a significant role in myocardial IRI by regulating the opening of mPTP. Mitochondria in cardiomyocytes are highly dynamic network structures, and previous research has shown that during IRI, there is an increase in mitochondrial fission in myocardial cells, and excessive mitochondrial fission is also considered one of the important triggers of IRI (Sharp et al., 2014). Regarding the crucial protein Drp1 involved in mediating mitochondrial fission, researchers such as Ong Sang-Bing found that inhibiting mitochondrial fission by deactivating Drp1 in HL-1 cardiac muscle cells can reduce cell sensitivity to mPTP opening and play a cardioprotective role in IRI (Ong et al., 2010). Additionally, the MFN1-MFN2 complex located on MAMs is closely related to the process of mitochondrial fusion and fission. The reduced expression of MFN1 and MFN2 can alleviate IRI in mouse myocardium and reduce the infarct area (Hall et al., 2016).

### 6.3 Cardiomyocyte hypertrophy and heart failure

Heart failure represents the end-stage of cardiovascular disease, where factors such as hypertension, valvular heart disease, and aortic stenosis lead to increased physiological or pathological workload on the heart. This can lead to compensatory myocardial hypertrophy, marked by the enlargement of individual cardiac muscle cells rather than a rise in their quantity. This condition is often accompanied by pathological alterations, such as myocardial dysfunction and interstitial fibrosis, which can ultimately advance to heart failure. In the aforementioned pathological process, mitochondrial function and Ca^2+^ homeostasis play critical roles. Researchers like Gutierrez found that during myocardial hypertrophy induced by norepinephrine, there is an increased distance between the SR and mitochondria in myocardial cells, while mitochondrial calcium uptake decreases (Gutiérrez et al., 2014). This forces myocardial cells to rely on glycolysis for energy production due to reduced mitochondrial oxidative activity, thus promoting the development of myocardial hypertrophy. Furthermore, after reducing RYR2 in the hearts of cRyr2KO mice by gene knockout, it was found that the heart showed heart failure-related symptoms such as myocardial and fibrous hyperplasia (Bround et al., 2013). This also indicates that disruptions in Ca^2+^ homeostasis might be a significant feature of heart failure. Deviations in mitochondrial calcium uptake have been consistently observed in both aging and chronic myocardial cell failure. Mitochondrial calcium levels significantly increase in aged mice and patients, accompanied by the accumulation of glycation products. Building upon the study by Ruiz-Meana et al. (Ruiz-Meana et al., 2019), this research reinforces the idea that RyR2, a protein located within the sarcoplasmic reticulum (SR) of aged mice, is highly susceptible to glycation, a process linked to aging. This glycation of RyR2 in heart muscle cells appears to contribute to age-related calcium leakage, which can ultimately damage mitochondria and contribute to various aging-related issues.

Recent research has revealed that MAMs, as essential channels for Ca^2+^ transfer between the SR and mitochondria, in which protein complexes such as FUNDC1, IP3R, MFN1/2, and Sig-1R also mediate the pathogenesis of heart failure. IP3R is a gated calcium channel located in the ER (SR) and plays a crucial role in myocardial responses to various stimuli that trigger hypertrophic reactions (Garcia and Boehning, 2017). FUNDC1, located in the OMM, binds to its isoform IP3R-2, mediating the transport of Ca^2+^ from the SR to the mitochondria and the cytoplasm. Studies suggest a reduction in FUNDC1 and SR-mitochondrial contacts in heart failure patients. Investigating the underlying regulatory mechanisms, research indicates that the FUNDC1/MAMs/CREB/Fis1 signaling pathway is significantly inhibited in the hearts of heart failure patients (Ren et al., 2020). Reduced FUNDC1 leads to decreased SR-mitochondrial connections, resulting in a significant decline in Ca^2+^ levels. The Ca^2+^-sensitive transcription factor CREB responds to changes in Ca^2+^ levels, suppressing Fis1 expression. This ultimately hinders mitochondrial fission, exacerbating heart dysfunction and heart failure. Additionally, studies have demonstrated significant reductions in mouse myocardial hypertrophy and secondary left ventricular functional impairment after administering Sig-1R agonists in mouse models of myocardial hypertrophy, such as abdominal aortic banding or TAC at the aortic arch (Bhuiyan et al., 2010; Tagashira et al., 2010). Sig-1R interacts with BiP to form a complex, regulating Ca^2+^ transport by stabilizing IP3R (Reane et al., 2020). Moreover, Sig-1R engages with RyR to form a complex, reducing Ca^2+^ leakage, ameliorating myocardial hypertrophy, and facilitating ATP synthesis (Tagashira et al., 2013). Furthermore, mitochondrial homeostasis is intricately linked to the progression of myocardial hypertrophy and heart failure. Studies in a guinea pig heart failure model revealed reduced levels of the mitochondrial homeostasis-related regulatory proteins MFN1 and MFN2 (Goh et al., 2016). Additionally, cardiac-specific MFN2 deficiency in mice results in a certain degree of myocardial hypertrophy (Papanicolaou et al., 2011).

### 6.4 Diabetic cardiomyopathy

Diabetic cardiomyopathy (DCM), first described by Rubler et al., refers to myocardial dysfunction independent of other factors such as valvular heart disease, hypertension, or coronary artery disease in diabetic patients (Rubler et al., 1972). The pathogenesis of DCM involves disturbances in glucose and lipid metabolism, oxidative stress, neurohumoral changes, cardiac metabolic abnormalities, and mitochondrial dysfunction (Hölscher et al., 2016; Ritchie and Abel, 2020). Disruption of calcium homeostasis and subsequent mitochondrial dysfunction are pivotal in myocardial cellular dysfunction in DCM, as stable Ca^2+^ dynamics are essential for myocardial cell excitation-contraction coupling and mitochondrial energy supply equilibrium (Bers, 2002; Gutiérrez et al., 2014). There is substantial evidence indicating that MAMs play a crucial role in DCM pathogenesis by regulating calcium homeostasis. FUNDC1, a highly conserved protein localized to mitochondria, is closely associated with maintaining the structure of MAMs and Ca^2+^ transport between the SR and mitochondria. Wu et al. were the first to report that diabetes can impact calcium homeostasis by promoting MAM formation, leading to mitochondrial dysfunction and DCM development (Wu et al., 2019). Under high-glucose conditions, AMP-activated protein kinase (Ampk) inactivation initiates FUNDC1. This results in excessive MAM formation, allowing an excessive influx of Ca2+ into mitochondria, ultimately leading to mitochondrial dysfunction and myocardial impairment. Simultaneously, FUNDC1 binds to IP3R2, inhibiting its ubiquitination and proteasomal degradation pathway, further promoting mitochondrial calcium overload. Similar alterations have been observed in normal myocardial cells through adenoviral FUNDC1 overexpression. Genetic deletion of FUNDC1 significantly improves high-glucose-induced MAM formation, thereby blocking regulatory mechanisms and reversing DCM progression. FUNDC1 emerges as a crucial target for DCM treatment. Furthermore, excessive ROS accumulation and cellular apoptosis are part of the pathological mechanisms underlying DCM. In DCM rat models, excessive ROS accumulation and ER stress lead to cellular apoptosis. ER stress markers PERK, IRE1, and ATF6 are activated in myocardial cells. Specific silencing of PERK on MAMs can prevent cellular apoptosis under high-glucose conditions (Liu et al., 2013). These studies demonstrate that MAMs play a pivotal role in DCM progression by mediating calcium homeostasis and subsequent mechanisms, including mitochondrial dysfunction, oxidative stress, and cellular apoptosis. This opens up new avenues and strategies for DCM treatment.

Disruptions in communication between the ER and mitochondria, mediated by specialized contact sites called MAMs, are implicated in the development of various cardiovascular diseases. MAMs play a critical role in regulating cellular mechanisms, and their dysfunction contributes to the pathological processes underlying atherosclerosis, ischemia-reperfusion injury, heart failure, and diabetic cardiomyopathy. The above studies have suggested that Ca^2+^may act as a messenger of intracellular information transmission. Therefore, Ca^2+^ homeostasis plays a crucial role in the pathogenesis of CVDs. For example, ER-mitochondrial tethering protein PACS2 has been linked to the OX-LDL-induced EC apoptosis owing to its regulatory effects on calcium overload in AS pathology. Similarly, protein complexes such as IP3R-Grp75-VDAC and MFN1-MFN2 can trigger myocardial IRI by inducing Ca^2+^ overload and over-opening of mPTP. In addition, Fundc1 was found to promote calcium overload by increasing the formation of MAMs, which further leads to mitochondrial dysfunction and cardiac dysfunction. Although excessive calcium is detrimental, insufficient Ca^2+^ levels can also damage various cardiovascular processes. Factors like increased distance between the SR and mitochondria, and decreased levels of FUNDC1 protein in cardiomyocytes, can decrease intracellular Ca^2+^ concentration. This forces heart muscle cells to rely more heavily on glycolysis for energy production, compromising mitochondrial function. Consequently, this metabolic shift can promote the development of myocardial hypertrophy and heart failure. These findings highlight the significance of MAMs-mediated calcium homeostasis in mitochondrial function and pathophysiology of CVDs, and further suggest that regulating MAMs-mediated calcium homeostasis may be a new strategy for treating age-related CVDs.

## 7 Discussion and perspectives

This article delves into the pathophysiological functions of mitochondria and the ER, elucidating the structural composition and functional mechanisms of their physical connection through MAMs. Furthermore, it explores research advancements in the context of MAMs concerning age-related CVDs. MAMs, acting as a bridge for communication between mitochondria and the ER, play a crucial role in regulating various aspects, including Ca^2+^ transport, mitochondrial dynamics, lipid synthesis and transport, autophagy, ER stress, inflammation, and cell apoptosis. All these processes are intimately linked to the onset and progression of CVDs. Furthermore, these pathological mechanisms gain distinctive significance in the aging process. In aging cells, the functionality of organelles such as mitochondria and the ER gradually deteriorates compared to normal cells. Simultaneously, the accumulation of a significant amount of ROS in aging organisms leads to the formation of an oxidative stress environment, impacting the progression of CVDs. Additionally, aging influences the arterial structure and function within the cardiovascular system, increasing the risk of CVDs.

With the ongoing progression of population aging, the incidence of related CVDs is steadily increasing, highlighting the urgent need for more effective treatment and intervention methods. In the context of age-related CVDs, MAMs play a dual role. They mediate oxidative stress responses of aging by participating in ROS production and regulate the pathological progression of CVDs such as atherosclerosis, IRI, myocardial cell hypertrophy, and DCM. Hence, proactive research into the regulatory proteins within MAMs holds significant importance for the treatment of CVDs. However, the signal regulation mechanisms of MAMs have not been fully elucidated at present. Future research should further explore the intricate mechanisms by which MAMs regulate cardiovascular health, particularly in the context of aging. This understanding could pave the way for the development of novel therapeutic strategies for CVDs in the aging population. Such strategies could involve biologics targeting MAM-related proteins, offering new and potentially more effective avenues for disease management.
